# The rheumatoid foot: a systematic literature review of patient-reported outcome measures

**DOI:** 10.1186/1757-1146-3-12

**Published:** 2010-07-09

**Authors:** Steven Walmsley, Anita E Williams, Mike Ravey, Andrea Graham

**Affiliations:** 1 Directorate of Prosthetics, Orthotics and Podiatry and Centre for Rehabilitation and Human Performance Research, University of Salford, Greater Manchester, UK; 2 Centre for Nursing, Midwifery and Collaborative Research, University of Salford, Greater Manchester, UK

## Abstract

**Background:**

The foot is often the first area of the body to be systematically affected by rheumatoid arthritis. The multidimensional consequences of foot problems for patients can be subjectively evaluated using patient-reported outcome measures (PROMs). However, there is currently no systematic review which has focused specifically upon the PROMs available for the foot with rheumatoid arthritis. The aim of this systematic review was to appraise the foot-specific PROMs available for the assessment and/or evaluation of the foot affected with rheumatoid arthritis.

**Methods:**

A systematic search of databases was conducted according to pre-defined inclusion/exclusion criteria. PROMs identified were reviewed in terms of: conceptual bases, quality of construction, measurement aims and evidence to support their measurement properties.

**Results:**

A total of 11 PROMs were identified and 5 papers that provided evidence for the measurement properties of some of the PROMs. Only one of the PROMs was found to be RA disease-specific. The quality of construction, pretesting and presence of evidence for their measurement properties was found to be highly variable. Conceptual bases of many of the PROMs was either restricted or based on reductionist biomedical models. All of the PROMs were found to consist of fixed scales.

**Conclusions:**

There is a need to develop an RA-disease and foot-specific PROM with a greater emphasis on a biopsychosocial conceptual basis, cognitive pre-testing methods, patient preference-based qualities and evidence to support the full complement of measurement properties.

## Background

The foot is often the first area of the body to be systematically affected by rheumatoid arthritis (RA) [1,2]. Upon diagnosis, approximately 16% of patients with RA have foot involvement [3], in 15% of cases the forefoot is the first area of the body to become symptomatic [4], and virtually 100% of patients report foot problems within 10 years of RA onset [5]. These clinical foot problems have a significant effect on the person's functional ability which is known to lead to important emotional experiences for patients, including anger and sadness [6].

The multidimensional consequences of foot problems can be subjectively assessed and evaluated using patient-reported outcome measures (PROMs). PROMs record patients' perspectives of their health, illnesses and the impact of any clinical interventions in a valid, reliable and feasible way [7]. They are an objective means of recording largely subjective outcomes and represent an ideal, economical and efficient method of measuring the quality and efficacy of care provided [8], integrating important psychosocial factors into a clinical assessment that otherwise may not be gathered.

According to Bowling [9], PROMs can be characterised in terms of their disease specificity (generic or non-disease specific), measurement objectives (discrimination, evaluation and prediction) and what they intend to measure (quality of life, health related quality of life (HrQoL) or health status).

The development of a PROM and establishment of its measurement properties most commonly entails the use of psychometric theory, which can be divided into two main methodological approaches: Classical Test Theory (CTT) and Item Response Theory (IRT) [10]. CTT (referred to as Traditional Psychometrics) utilises both item and sample statistics and is based upon 3 concepts, together known as True Score Theory [11]: test/observed score, true score and error score. Item response theory (known as Modern Psychometrics), on the other hand, utilises logistic response models to apportion individual items to the constructs of interest using conditional methods, according to their individual difficulty and the ability of subjects to respond positively to the items [12].

In selecting a PROM for use in either a clinical or research environment, the decision should be made upon its conceptual basis [13], the appropriateness of the PROM for the intended purpose and evidence for its measurement properties [14] (see Additional File 1, Table 1). A conceptual basis/model for a PROM is required to establish a well-defended rationale for and specify clearly the outcomes of interest for the instrument. The lack of an appropriate conceptual model for a PROM can result in a number of problems, including weak or incorrect scoring, analysis and interpretation of the data yielded [13].

Given the impact of foot problems for the adult with RA and the recognition that foot health interventions are an important aspect of health care for this patient group [15], a measurement of changes in foot health is vital to the monitoring of the foot and interventions for it from both the clinician's and the patient's perspective. This notion aligns with Darzi [16] who has recommended the use of PROMs, which focus on quality health care from a patient-centred perspective.

There are systematic reviews of PROMs for the foot and ankle in general [17] and for combined objective and subjective outcome measures, which includes a narrow selection of PROMs, with application for the foot with RA [18]. However, currently, there is no systematic review that has appraised specifically and in detail PROMs relevant for the foot with RA in terms of their conceptual bases, quality of construction and evidence for their measurement properties. Therefore, a systematic review of PROMs with relevance to the foot with RA is both timely and appropriate.

The aim of this systematic review was to appraise the quality of PROMs that may be used for the assessment and evaluation of the foot with RA in terms of their conceptual bases, quality of their construction, measurement aims and evidence for their measurement properties.

## Methods

This review was conducted using appropriate systematic review methods and is reported in accordance with the PRISMA statement [19]. A structured and exhaustive search of Pubmed, Embase, Cinahl, Ingenta, Science Direct and the Cochrane Collaboration Library was conducted on 15/10/2008 using the search terms: 'Rheumatoid arthritis' and 'foot index,' 'foot score,' 'foot instrument' and 'foot evaluation'. The search was restricted to publications in the English language. The reference lists of journal articles of interest were also searched and no restriction on year of publication was imposed to reduce publication bias.

PROMS selected for the review fulfilled the following inclusion criteria:

• Foot region specificity.

• Measurement of constructs relevant to the foot with RA, such as pain or activity limitation.

• Potential for application of the PROM in a research and/or clinical environment.

An additional search for specific evidence for the measurement properties of the PROMs was conducted by using the same search strategy, using the name of the PROM and 'measurement properties,' 'reliability,' 'validity,' 'validation,' 'responsiveness' and 'interpretability' as search terms.

All PROMs included in the review were appraised according to several pre-defined quality assessment criteria, including: The Scientific Advisory Committee of the Medical Outcomes Trust [20], The Patient-Reported Health Instruments Group [21] and the NHS Technology Assessment Board [7].

## Results

**Figure 1:**
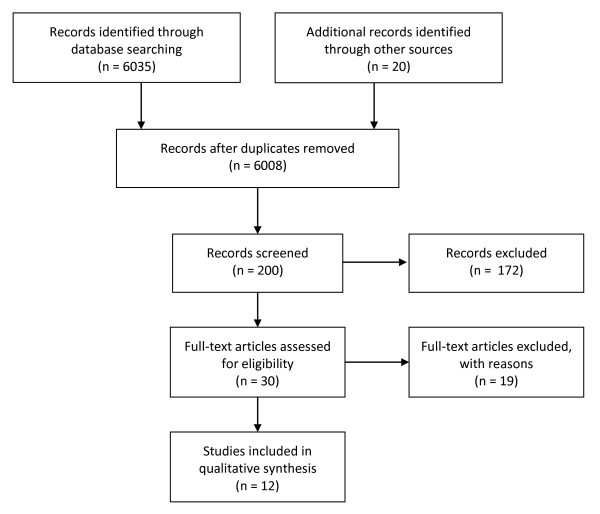
**Papers screened, identified and selected for review**.

Eleven PROMs were found to be eligible for inclusion in this review (Figure 1). Five papers that contributed evidence for the measurement properties for some of the 11 PROMs were identified and also included in this review. Of the 11 PROMs identified, 7 of them were judged to be non-disease specific and based upon CTT, 2 of them non-disease specific and based upon IRT; one was juvenile idiopathic arthritis (JIA) disease-specific and based upon CTT; and one was RA disease-specific and based upon IRT. Therefore, the papers were subdivided into 4 sub-groups according to these differences and reviewed accordingly.

### Non-disease specific PROMs (Classical Test Theory-based)

#### The Foot Function Index (FFI)

The Foot Function Index [22] is an evaluative PROM providing a measure of pain and disability, over a time period of one week, arising from joint diseases associated with older populations (See Additional File 1, Table 2). It is based upon the conceptual hypothesis that pain and activity limitation are the main complaints associated with musculoskeletal problems of the foot. However, the PROM does not evaluate other valid and equally important potential constructs, such as footwear, participation restriction [6,23] and other biopsychosocial factors associated with chronic pain and reflected in the International Classification for Functioning, Disability and Health (ICF) [24].

Content generation for the PROM did not include patients as recommended by Rattray and Jones [25] suggesting poor evidence for content validity. The FFI has evidence for a number of measurement properties (See Additional File 1, Table 3), including responsiveness [26,27] and sensitivity.

#### The Manchester Foot Pain and Disability Questionnaire (MFPDQ)

The Manchester Foot Pain and Disability Questionnaire [28] is an evaluative and discriminative PROM (See Additional File 1, Table 2) for identifying levels of foot pain and disability over the past month. However, with a conceptual basis that includes assessing foot pain and disability in terms of only the 3 constructs of pain intensity, activity limitation and personal appearance, the MFPDQ may not adequately capture the psychosocial experiences of patients arising from pain and disability in their feet [29]. Only 1 item is dedicated to the related issue of footwear.

Content generation for the MFPDQ entailed open interviews with patients with foot-related pain, disability, activity limitation and footwear problems, ensuring content validity. Proof to support the measurement properties of the MFPDQ is limited (See Additional File 1, Table 3).

#### The Podiatry Health Questionnaire (PHQ)

The Podiatry Health Questionnaire [30] is a discriminative and evaluative PROM (See Additional File 1, Table 2), for the assessment of foot-related HrQoL of podiatry patients with a range of foot conditions and the effectiveness of foot interventions over no specific time frame. Although the 7 constructs that form the conceptual framework of the PROM appear to be eclectic, each is represented only by one item, restricting the amount of information that can be gathered for each construct and increasing the potential for measurement error [31]. The PHQ has no evidence to support any of the measurement properties (See Additional File 1, Table 3).

#### The Bristol Foot Score (BFS)

The Bristol Foot Score (BFS) is an evaluative and discriminative PROM [32] that assesses the impact of foot problems on everyday life from the patient's perspective over 2 weeks, in terms of HrQoL (See Additional File 1, Table 2). The constructs measured by the BFS can be considered to provide a reasonably comprehensive coverage of HrQoL [33].

Content for the BFS was generated through interviews with patients with a wide range of conditions, such as RA, diabetes and osteoarthritis, ensuring that the items are highly relevant to patients and providing evidence for content validity. However, no experts were involved or a literature survey carried out, potentially restricting the breadth of content of the items generated [12]. There is no evidence available to support the majority of measurement properties for the PROM (See Additional File 1, Table 3).

#### The Foot Health Status Questionnaire (FHSQ)

The Foot Health Status Questionnaire is an evaluative and discriminative PROM [34] initially developed to have good clinical utility and psychometric soundness for the assessment of foot health status of patients both pre- and post-surgery (See Additional File 1, Table 2), over a period of one week. It was later recommended suitable for the assessment of general foot health status and the efficacy of non-surgical interventions. The four constructs measured by the FHSQ can be considered almost fully representative of the concept of health status [33]. Furthermore, the addition of a footwear subscale to the PROM helps to ensure comprehensive coverage of health status issues in relation to musculoskeletal conditions.

Content generation involved an unspecified number of focus groups with experts only, suggesting restricted content validity. The FHSQ has evidence for the majority of measurement properties (See Additional File 1, Table 3), including responsiveness [27] and clinical interpretability [35].

#### American Academy of Orthopaedic Surgeons Lower Limb Outcomes Assessment Instruments: Foot and Ankle Module (FAM)

The aim of the FAM [36] was to standardise treatment outcomes for various musculoskeletal conditions (ranging from acute trauma to chronic diseases such as RA) with respect to evaluation of symptoms from the patient's perspective. It is comprised of two individual scales: The Global Foot and Ankle Scale (GFAS), which refers to the past two weeks, and the Shoe Comfort Scale (SCS), which refers to no particular time frame (See Additional File 1, Table 2).

The constructs assessed by both the SCS and GFAS may be considered restricted for the purposes of the PROM. With a conceptual basis that considers only structure and function, the GFAS does not consider relevant concepts such as health status and HrQoL and thus the impact of musculoskeletal foot conditions from a biopsychosocial perspective. With respect to the SCS, the items are dedicated only as to whether patients can wear different types of footwear, not a subjective evaluation as to whether their footwear is deemed comfortable or not. A study comparing patient perceptions of footwear for patients with either RA or diabetes, [37] demonstrated that footwear comfort and appearance are particularly important to patients with RA.

Content generation for the FAM did not involve interviews with patients, potentially restricting content validity [38]. Further to this, it lacks evidence to support the majority of measurement properties, with the exception of clinical interpretability [39], internal consistency and test-retest reliability, face validity and clinician feasibility (See Additional File 1, Table 3).

#### The Rowan Foot Pain Assessment Questionnaire (ROFPAQ)

The Rowan Foot Pain Assessment Questionnaire [40], represents an attempt to provide a PROM that evaluates the multidimensional aspects of chronic foot pain over no specific time period (See Additional File 1, Table 2). It was developed with reference to the Gate Control Theory of Pain [41]. Conceptually, the ROFPAQ may be considered too restricted as concepts such as HrQoL, health status, and patient experiences of chronic disease span far beyond pain [42].

Content generation for the ROFPAQ entailed 6 focus groups and 2 semi-structured interviews with patients, ensuring good evidence of content validity, and the PROM has evidence to support many of the measurement properties (See Additional File 1, Table 3).

### Disease-specific PROM (Classical Test Theory-based)

#### The Juvenile Arthritis Foot Disability Index (JAFI)

The Juvenile Arthritis Foot Disability Index [43] is an evaluative and discriminative PROM. It was designed for the assessment of foot-related disability in children and adolescents with juvenile idiopathic arthritis (juvenile RA) in terms of severity and the effectiveness of interventions, for a period of one week (See Additional File 1, Table 4).

The authors ensured a good conceptual basis by structuring the subscales of the PROM according to the ICF [44]. However, with only 1 item enquiring about the availability of shoes, the authors greatly under-represent the importance of footwear [45]. Content generation for the JAFI involved two experts and review of the items present in the FFI [22] and Sundbom Arthritis Foot Evaluation Index (SAFE) [46]. However, the SAFE has not undergone validation, and the use of only 2 experts in generating content for the JAFI does not suggest evidence for content validity. The JAFI has very limited evidence to support its measurement properties (See Additional File 1, Table 5).

#### Non-disease specific PROMs (Item Response Theory-based)

##### The Revised Foot Function Index

The Revised Foot Function Index (FFI-R) was developed by Budiman-Mak *et al *[47] to address their perceived limitations of the original FFI [22] and exists in both a long (FFI-RL) and short (FFI-RS) form format (See Additional File 1, Table 4). Development of the FFI-R was allied closely with the ICF [44] and the authors acknowledged the importance of footwear, generating 10 items dedicated to the issue in terms of pain, function and psychosocial consequences. Thus, conceptually, the FFI-R can be considered a reasonably comprehensive measure of foot HrQoL [29]. Although content generation for the FFI-R was varied in approach, no patients were directly involved in the item generation process, potentially restricting content validity [25].

The FFI-R was developed using a 1-parameter IRT model, known as the Andrich Rating Scale (ARS) model [47], for item reduction and establishment of the subscales of the FFI-RL. In order for the (sub) scales of a PROM to be fitted to a 1-parameter model, it is imperative that the (sub) scales fitted can demonstrate uni-dimensionality and item local independence [48]. There was no evidence to demonstrate these pre-requisite properties, suggesting that the FFI-R was not successfully fitted to the ARS. Both the FFI-RS and FFI-RL have no evidence to support measurement properties other than for face and content validity (See Additional File 1, Table 5)

##### The Foot and Ankle Ability Measure (FAAM)

The Foot and Ankle Ability Measure [49] is an evaluative and discriminative PROM developed to permit the assessment of individuals with a wide range of musculoskeletal conditions affecting their foot, ankle and lower limb in terms of physical performance (See Additional File 1, Table 4), over a week. The conceptual basis of the FAAM is based solely on the structure and function component of the ICF [44], and thus there is no acknowledgement of the importance of biopsychosocial factors within the ICF [50]. Content generation for the FAAM entailed review of the literature of musculoskeletal conditions of the foot and lower limb, followed by consultation with both experts and patients regarding important items to consider, which suggests good content validity.

Both the subscales of the FAAM were fitted to a 2-parameter IRT model (Bond and Fox, 2007). Procedures were implemented to ensure that the FAAM subscales demonstrated the necessary conditions of uni-dimensionality and item independence. The FAAM has evidence for the vast majority of measurement properties [51] (See Additional File 1, Table 5).

#### Disease-specific PROM (Item Response Theory-based)

##### Leeds Foot Impact Scale (LFIS)

The Leeds Foot Impact Scale [52] is an evaluative and discriminative PROM developed specifically to assess the foot with RA (See Additional File 1, Table 4) with demonstrable measurement properties and wide applicability for the evaluation of the effectiveness of foot health interventions, in both research and clinical environments. The constructs assessed by the LFIS are closely associated with the domains of the ICF [44], creating a strong conceptual basis, and a strong emphasis is placed upon the importance of footwear. The PROM focuses upon the qualitative aspects of pain, stiffness and biopsychosocial experiences arising from the foot with RA, including items that allow patients to convey associated feelings of depression, anxiety and social isolation. Content generation for the LFIS involved semi-structured interviews with 30 patients to ensure content validity.

The authors used a dichotomous IRT (Rasch) model [48], but no procedures were employed to ensure the condition of unidimensionality of the both subscales of the PROM prior to fitting, which is strongly recommended [48,53]. Moreover, the items for impairments and shoes/footwear, and for activities and participation are merged together resulting in the two subscales for the LFIS. The authors then claim to have successfully fitted these to the Rasch model. However, attempting to fit scales that measure multiple constructs violates the uni-dimensional assumptions necessary for ensuring that data fit to a Rasch model. This confuses the traits that the PROM is trying to measure and, ultimately, what the scores represent [48].

The LFIS has evidence to support some of its measurement properties (See Additional File 1, Table 5).

## Discussion

This systematic review identified 11 PROMs with potential application for the assessment and/or evaluation of the foot with RA, 9 of which are based on CTT and 3 on IRT. However, only 1 of the PROMs is RA disease-specific, 1 JIA disease specific and the other 8 are generic. For the assessment and evaluation of a specific disease condition, generic PROMs may lack sufficient levels of validity, responsiveness and sensitivity [12].

All of the PROMs vary in terms of the conceptual bases for their development, quality of the methodological procedures used for their development (item generation, selection and appraisal) and the amount of evidence available to support their measurement properties. In terms of their conceptual bases, only 5 of the PROMs identified can be considered to evaluate or assess musculoskeletal conditions of the foot from a biopsychosocial perspective [22,32,34,43,52], one of them is RA disease-specific [52] and the other JIA disease-specific [43]. The other PROMs identified are formulated on conceptual bases that are either very restricted, such as the ROFPAQ [40] with the sole assessment of pain, or rooted in reductionist biomedical models of disease, such as the FAM [36] with its strong emphasis on structure and function.

Biomedical models, such as the International Classification for Impairments, Disability and Handicaps [54], assume a linear progression from a health condition to impairments and disability [55] and do not take account of the bi-directional implications of environmental or personal factors, leading to an over-simplistic appraisal of the implications of chronic diseases for patients [56]. PROMs that use biomedical models their conceptual bases may lack a wide enough conceptual scope to comprehensively evaluate the impact of chronic conditions on feet, such as RA. Furthermore, only 5 of the PROMs identified [32,34,36,47,52] consider the implications of footwear for patients with musculoskeletal conditions of their feet, with only one of them RA disease-specific. Given the importance of footwear for the management of musculoskeletal conditions of the foot [57], particularly RA [15,58,59], it is an important omission.

The content generation methods employed for the development of each of the PROMs vary in terms of both approaches and quality. Rattray and Jones [25] argue that the generation of content for PROMs should entail a variety of sources, particularly the engagement of patients, to ensure that the items generated have content validity and are as relevant to patients as possible. Although many PROMs employed heterogeneous content generation methods, only 4 PROMs identified [28,32,40,52] used interviews with patients. It is known that patient and clinician perspectives of what is important are different [60,61], so those PROMS relying on experts only to generate content may have restricted relevance to patients.

Levels of pre-testing of the PROMs appears highly variable and, in some cases, absent from the methodological approaches used to develop them. Pre-testing of PROMs is necessary to ensure that all of the questions are consistently easy to read and understand by all respondents. This is to reduce measurement error and non-response by achieving what Groves and colleagues [62] suggest to be content, cognitive and usability standards. Such standards can be evaluated during the pre-testing phase via expert reviews, focus group discussions, cognitive interviews and field pre-testing [63]. Although many of the PROMs involved pre-testing of their content in terms of their content standards and usability standards, such as the FAM [36] and BFS [32], none of the PROMs involved pre-testing to ensure that they satisfy cognitive standards. This is evidenced by the FAAM [49] and LFIS [52], which feature questions containing complex terminology and potentially distressing words, respectively.

Evidence for the measurement properties of the PROMs is highly variable. Either no attempt has been made to establish evidence for a particular measurement property or procedures have been employed to demonstrate particular measurement properties that are incorrect or inappropriate. Furthermore, when considering the evidence for the measurement properties of PROMs based upon CTT, it should be realised that the procedures involved are both sample and context-dependent. This means that the evidence for the measurement properties has restricted validity for use on populations that differ from those used to develop evidence for the PROM. As most of the generic CTT-based PROMs did not include patients with RA in the development of their measurement properties, it can be considered that they have limited clinical and research utility for the assessment and evaluation of patients with feet with RA.

All of the PROMs identified consist of fixed scales, presenting every patient with the same set of items irrespective of their RA disease duration, severity and particular lifestyle. However, patients are influenced by the symptoms of RA to varying extents, depending upon the level of disease activity and duration [60] and environmental and patient characteristics [64]. Thus, a PROM consisting of fixed items that assume equal importance and relevance for every patient may not be the most appropriate or patient-centred means of assessing the impact of foot problems on people with RA. Although measuring the same outcomes for groups of patients may be advantageous and necessary for research such as clinical trials, for informing decision making in clinical practice, assessment of change unique to the individual has been considered to be more beneficial [65]. This is possible through the use of patient preference-based questionnaires, which are geared towards the assessment of the specific individual and can permit the measurement of concepts such as individual health-related quality of life [66].

Patient preference-based questionnaires have been implemented and are currently used with considerable success [67]. These include the Patient Generated Index [68] and the MacMaster-Toronto Arthritis Patient Preference Questionnaire [69]. However, this systematic review could not identify RA or generic foot-specific idiographic PROMs with either the potential for use in the assessment of the foot with RA, or with any relevance to the foot.

Although this review has attempted to present a comprehensive appraisal and review of all foot-specific PROMs that are relevant for the assessment and/or evaluation of the foot with RA in both research and clinical environments, it should be realised that new PROMs may have been developed or additional evidence for the measurement of existing PROMs presented since the literature search for this review was conducted, Further, it was not possible to present the results of the literature search to experts and special interest groups to gauge its comprehensiveness prior to conducting the review. However, the findings have been scrutinised for accuracy and relevancy by two academic podiatrists and one independent academic. Despite this slight weakness in approach, this review has attempted to present a comprehensive appraisal and review of all foot-specific PROMs that are relevant for the assessment of the foot with RA in both research and clinical environments.

## Conclusions

PROMs should be embedded in the ethos of patient-centred quality health care [15]. Due to the impact of foot problems in patients with RA on HrQol, it is vital that a PROM is developed for the assessment and evaluation of the foot with RA that has a conceptual basis based upon the WHO [44] biopsychosocial model of chronic diseases. Further to this, it should be adequately pre-tested, have evidence to support all of the measurement properties and feature the capacity for patient preference-based assessment.

## Competing interests

The authors declare that they have no competing interests.

## Authors' contributions

SW conceived the study design, conducted the systematic review, interpreted the findings and drafted the manuscript. AEW, MR and AG reviewed the manuscript and provided academic support throughout. All authors read and approved the final manuscript.

## Supplementary Material

Additional file 1 **Tables 1-5**. Table 1: Measurement properties required for descriptive and evaluative PROMs. Table 2: Description of development and content of Classical Test-Theory-based, generic, foot-specific PROMS. Table 3: Evidence for the scientific measurement properties of the CTT - based, generic, foot specific PROMs. Table 4: Description of development and content of Classical Test Theory and Item Response Theory-based, JIA diseasespecific, RA disease-specific and generic foot-specific PROMs. Table 5. Evidence for the measurement properties of the Classical Test Theory and Item Response. Theory-based, generic andJIA disease-specific and RA disease-specific, foot - specific PROMs. Click here for file
