# Supplementary material for: The rheumatoid foot: a systematic literature review of patient-reported outcome measures
Source: J Foot Ankle Res. 2010 Jul 9;3:12. doi: 10.1186/1757-1146-3-12 (PMC2914720; doi:10.1186/1757-1146-3-12)
Supplement: Additional file 1 — Tables 1-5. Table 1: Measurement properties required for descriptive and evaluative PROMs. Table 2: Description of development and content of Classical Test-Theory-based, generic, foot-specific PROMS. Table 3: Evidence for the scientific measurement properties of the CTT - based, generic, foot specific PROMs. Table 4: Description of development and content of Classical Test Theory and Item Response Theory-based, JIA diseasespecific, RA disease-specific and generic foot-specific PROMs. Table 5. Evidence for the measurement properties of the Classical Test Theory and Item Response. Theory-based, generic andJIA disease-specific and RA disease-specific, foot - specific PROMs. [file 1757-1146-3-12-S1.PDF]

Table 1. Measurement properties required for descriptive and evaluative PROMs.

Assessment criteria		Description	Example(s) of criteria concerned
**Conceptual basis and measurement structure**		What the PROM specifically aims to measure and its intended population.	Appropriate scale and subscale structure. Suitable scoring with an appropriate level of measurement (nominal, ordinal, interval or ratio).
**Measurement Properties**	**Reliability**	The degree to which a PROM is free of random error and reflects the true score. In terms of psychometric theory C/o **Internal consistency** (the level of homogeneity of the items in the scale and thus whether they are all measuring the construct of interest) and **Test-retest reliability** (its temporal stability).	**Internal consistency**- Cronbach's α or split half reliability .**Test-retest reliability** - intraclass correlation co-efficients or Pearson's Product Moment Correlation Co-efficients to assess the level of agreement between 2 administrations.
	**Validity**	How well the PROM measures what it is intended to measure. C/o **Face validity** concerns whether it appears to be measuring the construct(s) of interest; **content validity** the extent to which the items satisfy the entirety of the concept(s) being measured; **internal construct validity** whether there is evidence available to support its theoretical basis; **external construct validity** - interpretation of the scores from what is being measured; and **criterion validity** regards how well the findings from the new scale correlate with any existing gold standards.	**Content validity** - assessed through expert and patient opinion regarding relevance of and breadth of coverage of the items of the PROM. **Construct validity -** ascertained through hypothesis testing and the development of correlates for predictive, convergent and divergent validity. **Criterion validity** - obtained through the level(s) of correlation with existing gold standard(s).
	**Responsiveness**	Ability of the PROM to detect change over time that is of clinical relevance to the intended population(s).	No general consensus exists regarding responsiveness assessment, but can include distribution based methods (effect size, standardised response mean) or anchor based methods.
	**Precision**	Capability of the PROM to discriminate effectively between patients in terms of their reported condition and therefore the range of responses permitted.	Item coverage of the defined constructs of the PROM, the number of response categories (thus level of measurement) and the presence of any end effects (floor and ceiling) are important considerations.
	**Interpretability**	What the quantitative changes in instrument scores actually mean to both patients and clinicians.	Anchor and distribution based methods are available, such as score distribution and health transition items, respectively.
**Practical properties**	**Clinician feasibility and Patient acceptability**	The time and effort required by both patients and clinicians - includes both acceptability (pertains to how prepared patients and clinicians are to use the PROM) and feasibility (how easy the PROM is to complete. costs and time demands).	Acceptability and feasibility can be assessed using techniques such as collation of patient and clinician opinions, response rates, missing values and extent of instrument completion.

Table 2. Description of development and content of Classical Test-Theory-based, generic, foot-specific PROMS.

	PROM	Authors	Content generation methods	Items	Type of response scale	Constructs assessed
**Classical test theory-based, generic, foot specific PROMS**	The Foot Function Index [22]	Budiman-Mak *et al*, (1991)	Not stated	23	10 cm VAS	Foot Pain<br>Foot-related Disability<br>Foot-related Activity Limitation
	The Manchester Foot Pain and Disability Questionnaire [28]	Garrow *et al*, (2000)	Open-ended interviews with 32 patients attending foot clinics with foot-related pain, disability, activity limitation and footwear problems	19	3 point adjectival rating scale	Functional limitation<br>Pain intensity<br>Personal appearance
	The Podiatry Health Questionnaire [30]	Macran *et al*, (2003)	Unspecified number of podiatry managers and podiatrists	7	6 point adjectival rating scale<br>20 cm VAS	Walking/getting about<br>Hygiene<br>Nail care<br>Foot pain<br>Worry/concern<br>Quality of life
	The Bristol Foot Score [32]	Barnett *et al*, (2005)	Semi-structured interviews with 10 patients with unspecified foot problems (7 females, 3 males)	15	3 to 6 point adjectival rating scales	Concern and pain<br>Footwear and general foot health<br>Mobility
	The Foot Health Status Questionnaire [34]	Bennett *et al* (1998)	Unspecified number of podiatric surgeons	13	5 point adjectival rating scale	Foot pain<br>Foot function<br>Footwear<br>General Foot Health
	The AAOS Lower Limb Outcomes Assessment Instrument: Global Foot and Ankle Scale [36]	Johanson *et al*, (2004)	Unspecified number of focus groups with content-knowledgeable experts<br>Literature review of potentially relevant outcome measures	20	5 to 7 point adjectival rating scale<br>Guttman scale	Pain<br>Function<br>Stiffness<br>Swelling<br>Giving way
	The AAOS Lower Limb Outcomes Assessment Instrument: Shoe Comfort Scale [36]	Johanson *et al*, (2004)	Focus groups with content-knowledgeable experts<br>Literature review of potentially relevant outcome measures	5	3 point adjectival rating scale	Ability to wear different kinds of footwear
	The Rowan Foot Pain Assessment Questionnaire [40]	Rowan, (2000)	6 focus groups and 2 semi-structured interviews with patients with chronic foot pain (17 females, 5 males)	39	5 point adjectival rating scale	Sensory pain<br>Affective pain<br>Cognitive dimensions of pain<br>Questionnaire Comprehension

Table 3. Evidence for the scientific measurement properties of the CTT – based, generic, foot specific PROMs.

Type of PROM	Instrument and Author(s)	Measurement Properties of CTT-based generic PSRQ										Practical properties	
		Reliability		Validity					Other key properties				
		Internal consistency	Temporal stability	Content validity	Face validity	Criterion validity	Construct validity		Responsiveness	Sensitivity	Clinical interpretability	Patient acceptability	Clinician feasibility
							Internal	External					
Classical test theory-based, generic, foot specific PROMS	FFI	✓	✓					✓	✓	✓		✓	✓
	MFPDQ			✓	✓			✓				✓	
	PHQ												
	BFS		✓	✓	✓							✓	✓
	FHSQ	✓	✓		✓		✓		✓	✓	✓	✓	
	FAM	✓	✓		✓						✓		✓
	ROFPAQ	✓	✓	✓	✓			✓		✓		✓	✓

FFI = Foot Function Index [22]; MFPDQ = Manchester Foot Pain and Disability Questionnaire [28]; PHQ = Podiatry Health Questionnaire [30]; BFS = Bristol Foot Score [32]; FHSQ = Foot Health Status Questionnaire [34]; FAM = Foot and Ankle Module [36]; ROFPAQ = Roland Foot Pain Assessment Questionnaire [40]. ✓ = Evidence available

Table 4. Description of development and content of Classical Test Theory and Item Response Theory-based, JIA disease-specific, RA disease-specific and generic foot-specific PROMS.

	PROM	Authors	Content generation methods	Total number of items	Type of response scale	Constructs assessed
**Classical test theory-based, JIA disease and foot specific PROMs**	The Juvenile Arthritis Foot Disability Index [43]	Andre *et al*, (2004)	Interviews with 2 content-knowledgeable physiotherapists<br>Review of items in 2 potentially relevant foot-specific outcome measures: the Foot Function Index and Sundbom Arthritis Foot Evaluation Index.	23	10 cm VAS	Foot Pain<br>Foot-related Disability<br>Foot-related Activity Limitation
**Item response theory-based, generic and foot specific PROMs**	The Revised Foot Function Index – Long Form [47]	Budiman-Mak *et al*, 2006	Review of the original items of the FFI<br>Literature review of mobility in elderly people<br>Interviews with an unspecified number of content knowledgeable clinicians	68	6 point adjectival rating scale	Foot pain<br>Stiffness<br>Difficulty related to foot function<br>Activity limitation<br>Social functioning
	The Revised Foot Function Index – Short Form [47]			34		Foot function summary
	The Foot and Ankle Ability Measure [50]	Martin, 2003	Literature review of signs, symptoms and limitations in physical function associated with musculoskeletal disorders of the foot, ankle and lower limb.	29	5 point adjectival rating scale	Activities of daily living<br>Sports
**Item response theory-based, foot and disease-specific PROMs**	The Leeds Foot Impact Scale [52]	Helliwell *et al*, (2005)	Semi-structured interviews with 30 patients with RA-related foot problems	51	Binary categorical scale	Impairments/shoes<br>Activities/participation

Table 5. Evidence for the measurement properties of the Classical Test Theory and Item Response Theory-based, generic and JIA disease-specific and RA disease-specific, foot - specific PROMs.

Type of PROM	Instrument and Author (s)	Measurement Properties of CTT-based generic PSRQ										Practical properties	
		Reliability		Validity					Other key properties				
		Internal consistency	Temporal stability	Content validity	Face validity	Criterion validity	Construct validity		Responsiveness	Sensitivity	Clinical interpretability	Patient acceptability	Clinician feasibility
							Internal	External					
Classical test theory-based, JIA disease and foot specific PROMs	JAFI		✓		✓					✓			
Item response theory-based, generic and foot specific PROMs	FFI-RL			✓	✓								
	FFI-RS			✓	✓								
	FAAM		✓	✓	✓		✓	✓	✓	✓	✓		✓
Item response theory-based, foot and disease-specific PROMs	LFIS	✓	✓	✓	✓		✓						✓

JAFI = Juvenile Arthritis Foot Disability Index [43]; FFI-R = Revised Foot Function Index (FFI-RS = short scale, FFI-RL = long scale) [47]; FAAM = Foot and Ankle Ability Measure [49]; LFIS = Leeds Foot Impact Scale [52]. ✓ = Evidence available
